# High levels of alcohol intoxication and strong support for restrictive alcohol policies among music festival visitors

**DOI:** 10.1186/s13011-019-0203-8

**Published:** 2019-04-15

**Authors:** Kristin Feltmann, Tobias H. Elgán, Johanna Gripenberg

**Affiliations:** STAD (Stockholm Prevents Alcohol and Drug Problems), Centre for Psychiatry Research, Department of Clinical Neuroscience, Karolinska Institutet & Stockholm Health Care Services, Stockholm County Council, Norra Stationsgatan 69, 11364 Stockholm, Sweden

**Keywords:** Alcohol prevention, Large music event, Blood alcohol concentration (BAC), AUDIT-C, Alcohol intoxication, Alcohol drinking habits, Opinions on alcohol, Gender effects

## Abstract

**Background:**

Alcohol intoxication is associated with problems such as violence, injuries, drunk driving and sexual risk-taking, and music festivals are considered a high-risk setting for high levels of alcohol consumption. This study investigates intoxication levels, drinking habits, and opinions on alcohol use and alcohol policies among visitors at one of the largest music festivals in Sweden in 2017.

**Methods:**

A cross-sectional study assessing alcohol intoxication levels was conducted at a music festival (~ 50,000 attendees). Two research teams collected data at the two festival entrances during two nights, from approximately 6:00 pm to 01:30 am. Blood alcohol concentration (BAC) levels were measured using breath analyzers. A face-to-face questionnaire was used to interview attendees about their alcohol use in the past 12 months using the Alcohol Use Disorder Identification Test-Consumption (AUDIT-C), as well as about their personal opinions on alcohol use and alcohol policies (statement). BAC levels were compared between categories of various factors using Mann-Whitney U test and Kruskal-Wallis test. The distributions of BAC, AUDIT-C and statement category across gender was analyzed using Pearson’s Chi-square tests. Associations of BAC levels with different factors were analyzed using Spearman rank correlation and multinomial logistic regression.

**Results:**

A total of 1663 attendees were randomly selected and invited to participate, and 1410 consented (63.7% men, 34.9% women, age 16–64 years). The proportion of drinkers was 81%. Among the drinkers, the median BAC level was 0.082%. Thirty-one percent of the participants had a BAC level above 0.10%. Forty-two percent of the participants reported binge drinking monthly, and 20% said that they binge drank weekly. Sixty-three percent of participants reported risky drinking habits. A self-reported risky alcohol habit increased the risk of a high alcohol intoxication level at the festival. Respondents were supportive of restrictive alcohol policies. Men had significantly higher BAC levels, reported more often risky alcohol habits and were less supportive of restrictive alcohol policies than women.

**Conclusions:**

The results indicate that participants at music festivals in Sweden have high levels of alcohol intoxication and largely support restrictive alcohol policies. Thus, there is both a need and support for the implementation of alcohol prevention strategies at festivals.

## Background

Large music events, such as music festivals, attract large numbers of visitors. Examples include the music festivals Glastonbury (175,000 people) and Tomorrowland (185,000 people) in Europe and the Coachella Valley music and art festival (125,000 people) in the US. Music festivals are arenas where young people socialize, and alcohol drinking is often considered a part of the festival experience.

Alcohol drinking at these large events can have a number of negative consequences. First, alcohol consumption can cause various security problems that can harm both the individual who is drinking and bystanders, for example, drunk driving and resulting car accidents [1] and injuries inflicted by violent, alcohol-intoxicated individuals [2–4]. Furthermore, in the case of rapid evacuations due to fire alarms, bomb threats or terrorist attacks, heavily intoxicated individuals can slow or impede progress, thereby harming themselves or others. Simulation models by Moore and coworkers suggest that alcohol intoxication that hampers balance and increases staggering would cause a more disordered and slower crowd flow dynamic [5]. One example of how a disordered crowd flow can have tragic consequences was a large electronic dance music (EDM) street festival (with an estimated 1 million visitors) in Duisburg, Germany in 2010, at which 21 people suffocated and over 500 were injured. According to studies using video analysis, this tragedy was caused by overcrowdedness and by people falling, not crowd panic [6, 7].

Second, alcohol drinking increases sexual risk-taking [8–11], which can lead to the transmission of sexually transmitted diseases.

Third, sexual assaults are also associated with alcohol intoxication in both the victim [12] and the perpetrator [13–15]. In fact, in recent years, the media has frequently reported cases of sexual assaults and rape at large music festivals [16, 17]. The ‘me too’ movement has demonstrated that sexual assault is a widespread problem and that is not limited to any specific cultural setting [18–20]. The figures for such assaults might be particularly high at festivals [21–24], as many young women—the most common victims—gather at these events, where alcohol intoxication is usually high, and victims are more likely to report an assault to the police when they did not previously know the perpetrator [12].

Last, in addition to these acute risks of alcohol consumption, drinking large amounts of alcohol during late adolescence is associated with alcohol dependence later in life [25]. Furthermore, the numerous adverse health consequences of alcohol use represent a large socioeconomic burden [26, 27]. Hence, reducing alcohol use among young people is important for preventing acute and long-term alcohol-related problems.

Some studies have interviewed festival visitors about their alcohol drinking habits [28–30] and alcohol consumption at the festivals [31]. For example, about 30 % of visitors at Australian festivals in 2009 and 2011 reported binge drinking weekly [29, 30]. At a Scottish music festival in 2008, 88% of guests reported drinking alcohol at the festival, mainly alcohol they had brought themselves [31]. Moreover, participants also reported negative consequences due to alcohol drinking such as vomiting, heatstroke, unsafe sex, fighting and collapsing. Males were more likely to report drinking alcohol at the festival or to experience these negative consequences compared to females. Although there was a small increase in reporting alcohol drinking with age, age was rated as having a rather small influence [31]. Similarly, among Belgian club- and festivalgoers men reported to drink more frequently than women and more often before and after going out [28].

Few studies have measured alcohol intoxication levels in music festival settings. One example, a study at a large EDM festival in Miami (US), showed that 40% of blood samples were positive for alcohol, with an average blood alcohol concentration (BAC) level of approximately 0.10% but the effect of gender on BAC levels was not reported [32]. At a large Swedish 40-h EDM event held on a cruise ship, 94% had been drinking alcohol with an average BAC of 0.10%. Significantly more men than women had a BAC ≥0.10% [33]. Nevertheless, most studies have measured BAC levels in other nightlife settings, such as pub, bars and nightclubs and were conducted in several European and American cities [34–37]. Two of these studies reported that between 88 and 94% had detectable BAC levels with an average of approximately 0.10% [34, 35]. Together, both studies from festivals and clubs demonstrate a rather high average BAC level of 0.10%. Although the Swedish limit for driving under the influence (DUI) is 0.02%, at 0.10% the crime is considered to be gross. At a BAC level of 0.10%, a person starts to experience impairments in vision, speech, balance and motor control, perception and memory and is therefore defined as highly intoxicated in the present study. Furthermore, other studies conducted in clubs reported that a considerable proportion (between 28 and 70%) of participants had BAC levels higher than 0.08% [36, 37], the DUI level of the UK and some US states. Overall, these results emphasize the need for preventive interventions in nightlife settings.

Since 1996, our research group, STAD (Stockholm prevents alcohol and drug problems), has successfully developed, implemented and evaluated community-based multicomponent interventions targeting licensed premises to prevent alcohol- and drug-related problems. These interventions include community mobilization, staff training in ‘responsible beverage service’, and stricter law enforcement by the licensing board and the police [38]. Evaluations of these interventions have demonstrated a reduction in the level of alcohol-related violence [39–41] and overserving to intoxicated [42, 43] and underaged individuals [44]. Furthermore, calculations have demonstrated a societal cost-saving ratio of 1:39 due to a reduction in alcohol-related crimes and usage of health care [45]. This model has been disseminated to over 200 municipalities in Sweden [46] and has been adapted to other settings such as large sports arenas [47–49]. Moreover, within the European project ‘STAD in Europe’, the model is being adapted to other countries and settings, such as music festivals and public drinking.

Compared to events at licensed premises, such as pubs, bars and nightclubs, festivals are usually large events that stretch over several days. The majority of festival goers stay overnight in the adjacent camping area and do not need to drive home. Due to frequent traffic controls by the police and a low DUI limit of 0.02% BAC, many Swedish people abstain from drinking, if they need to drive. It is common to bring large quantities of alcohol to the camping area and consume these during the day and night; before, in between and after concerts. This camping culture together with the high prices of alcohol inside the festival area makes pre-loading highly likely. Therefore, we hypothesized that people who camp (e.g., using the camping entrance, holding a camping ticket) would be more likely to be highly intoxicated. Moreover, due to the large number of people and the vast festival area, control of alcohol consumption and alcohol-related problems is challenging. In addition, for the majority of the security or alcohol-serving staff, working at the festival is a temporary side-job, i.e., they are inexperienced and often untrained in responsible beverage service. Therefore, all components of the responsible beverage service intervention, such as training and control, need to be adapted to these specifics and challenges of this nightlife setting. Examples are a short and accessible form of education (e.g., online) and a better organized system of control in denying entrance to highly intoxicated individuals.

As part of the European project mentioned above, the current study aims to measure alcohol intoxication levels among attendees at one of the largest music festivals in Sweden. Specific research questions include the following: What proportion of attendees has consumed alcohol? What is the median BAC level among drinkers? What proportion of visitors has a high level of intoxication, defined as having a BAC level of 0.10% or more? What is the level of risky alcohol use among the attendees? Are there gender differences regarding alcohol consumption? Are BAC levels higher upon entering or exiting? What proportion of attendees supports restrictive alcohol policies? Which factors are associated with alcohol intoxication? Does camping increase the likelihood of being highly intoxicated? The findings will be used to assess whether an alcohol prevention intervention for large music events, such as music festivals, is needed.

## Materials and methods

### Design and setting

One of the largest, most popular music festivals in Sweden was chosen for data collection. This event is a 4-day outdoor festival attracting approximately 50,000 visitors each summer. Several popular international artists were performing at the festival the year the study was conducted (summer 2017), and the music genres included pop, rock, indie, and electronic music. Concerts started at 12 noon and ended at 2 am. Permission was obtained from the organizers and authorities to carry out the study at this specific event. The age limit to visit the festival was 13 years. A four-day festival ticket with and without camping cost approximately 300 and 260 US dollars, respectively.

In Sweden, the legal age to buy and consume alcohol at licensed premises is 18 years. At this event, people who were over 18 years old wore a color-coded bracelet indicating ‘over 18 years’. To obtain this bracelet visitors had to show their ID. Food, alcoholic and non-alcoholic beverages could then be purchased using the chip attached to their bracelet. It was possible to buy up to four alcoholic beverages per person per purchase, but the festival website informed that providing alcohol to minors would lead to exclusion from the festival and removal of the bracelet and that the incident would be reported to the police.

Most people arrived at the festival by car. However, the nearest tram station could be reached by a half-hour walk. There were eight camping areas, and bringing and consuming alcohol was allowed in all but one of these areas. The recommended age for camping was 16 years and over. Approximately 20,000 people could camp in the largest camping area. Alcohol could not be brought into the festival area from outside. Alcoholic beverages (e.g., beer, wine, hard liquor) were sold at four larger bar areas containing more than ten counters, five medium-sized bar areas containing up to ten counters and five smaller bars. One beer cost approximately eight US dollars. The purchased alcoholic beverages could be taken away from the serving area and consumed anywhere within the festival area.

The alcohol law in Sweden prohibits the serving of alcohol to underage people and obviously intoxicated individuals. Furthermore, the area where alcohol is served must be of a manageable size. Therefore, regular alcohol licenses prescribe that alcohol is served and consumed in a relatively well-defined area. However, in this case, alcohol could be consumed anywhere in the entire festival area due to an open alcohol permit. Hence, because of the size of the area (82,000 m^2^ ≈ 11 soccer fields) and the number of people who would be served at the festival, obtaining an overview of the area was not possible for serving staff. Because of this problem, the festival organizers employed approximately 40 ‘alcohol inspectors’, whose task was to identify intoxicated people, talk to them, remind them to eat and drink water frequently and if necessary, contact security staff for other actions, e.g., expulsion.

Furthermore, security staff was present to maintain order, search visitors’ bags for any beverages upon entering, prevent people who were highly intoxicated from entering and order highly intoxicated people to leave the festival area. Moreover, several police officers supervised the festival area.

The festival’s website encouraged safe drinking by calling for moderate alcohol consumption and reminding festivalgoers to drink water. Locations where drinking water (from taps) could be obtained free of charge (seven in total) could be found near all the restrooms and were indicated on the festival map. However, in contrast to many other licensed premises in Sweden, drinking water could not be obtained free of charge at the bars.

The media had reported on sexual assaults and rapes at the festival for several years. A few weeks after the festival ended, the police published the following data on this festival: 116 minor drug offenses, 24 reports of sexual assaults, five reports of rapes, one report of sexual coercion, 14 reports of violent assaults, ten cases of DUI near the festival area and 138 robberies/thefts. The number of sexual assaults (24 assaults among 50,000 visitors, i.e. 0.05%) seems to be at a higher rate than other festivals in Sweden that took place during the same year and were of comparable size (rates between 0.01 and 0.02% at festivals hosting 30,000–60,000 visitors). However, at a smaller festival hosting 6000 visitors, the rate was comparably higher (0.37%, 22 sexual assaults reported) [50].

### Outcome measurements

Data were collected through face-to-face interviews with questionnaires containing questions on demographics, alcohol use, and opinions about alcohol-related behaviors. The questions on demographics related to age, gender, and occupation. Regarding occupation, the following categories were used: working (full-time or part-time), studying (high school, university), unemployed or other occupation. The participants were also asked if they were about to enter or exit the festival and about the type of festival ticket they possessed (i.e., 1-day, 4-day, 4-day with access to a camping site).

Self-reported alcohol use within the last 12 months was assessed using the Alcohol Use Disorder Identification Test-Consumption (AUDIT-C). In a recent study, the AUDIT-C was psychometrically evaluated among 15–18-year-olds [51]. Results revealed a good internal consistency (alpha 0.75) and an excellent test-retest reliability (Intraclass Correlation Coefficient 0.93, however, an optimal cut-off score could not be determined. A review by Reinert and Allen suggests that the 3/4 cut-off of the AUDIT-C is more suitable for detecting hazardous drinking, while the 4/5 cut-off is more sensitive to alcohol use disorders [52]. Nevertheless, some studies have recommended the use of a cut-off of 6 for both genders [53, 54]. Data based on all three cut-offs have been presented to enhance the comparability of studies.

To explore opinions on alcohol use and alcohol policies the participants were to answer the following six statements: Statement 1, S1) A good night out means getting drunk. S2) It is acceptable for people under 18 to buy or be bought alcohol. S3) Drunk people ruin a night out. S4) Drunk people should be able to enter the festival area. S5) People who are drunk should not be able to obtain more alcohol. S6) Not providing people who are already drunk with more alcohol would improve nights out. Whereas statements S1 and S3 are related to opinions on alcohol use in relation to the nightlife experience, statements S2, S4, S5 and S6 are related to opinions on the alcohol policies based on the Swedish alcohol law on not serving alcohol to underage or obviously intoxicated people and not allowing obviously intoxicated people to be at licensed premises. To each statement participants were provided with the following seven answer options: Strongly agree, agree, neither agree or nor disagree, disagree, strongly disagree, do not know and prefer not to say.

Participants’ BAC levels were measured using breath analyzers (Dräger Alcotest 6820, Drägerwerk AG & Co. KGaA, Germany), which are used by the Swedish police authorities. Older models of this device have been validated through comparison to blood samples [55]. The device displays values corresponding to permille by mass in blood (mg/g, ‰), which are converted into and presented as a percent (g/dL, %).

### Procedure

Data were collected between 6:00 pm and 1:30 am on the second and third days of the four-day festival (Thursday and Friday). Two teams were used, each consisting of seven people working at one of the two entrances to the festival area so that both entrances were monitored simultaneously. Participants were recruited using the line method [56, 57]. Briefly, an imaginary line was drawn near the entrance. People passed the imaginary line when either entering or exiting the festival area. Upon entering, people had just been checked by security staff, and any liquids would have been taken from them. Every third person who crossed the line was asked to anonymously participate in the study. Upon refusal, the approximate age and gender were recorded. If the person recruited was part of a group, the whole group was invited to participate, and each member was assigned to one interviewer for simultaneous measurement insofar as possible. This approach was adopted based on indications of lower refusal rates in previous studies [58]. Upon agreeing to participate, each individual was assigned to a nearby interviewer. Since it was vital to maintain anonymity and confidentiality, no signatures were required from the participants for the informed consent process. The participants were first given a cup of water to rinse their mouths to obtain accurate readings from the alcohol breath test. The face-to-face interview was then performed, followed by the alcohol breath measurement. The result from the breath analyzer was instantly available on site and displayed to the participants if requested.

Visitors that reported an age below 16 years of age were denied participation in the study. No personal data were collected that would enable the identification of any individual.

### Statistical analysis

SPSS Statistics software (IBM Corporation, Version 24) was used to analyze the data in terms of descriptive analyses and to generate frequency and contingency tables. As the data (BAC, age) were not normally distributed, medians and interquartile ranges were reported, and the data were analyzed using the following nonparametric tests: Mann-Whitney U-test, Kruskal-Wallis test, and Spearman’s rank correlation. Frequencies of opinions on alcohol use and policies were presented as the percentage of people agreeing (sum of ‘strongly agree’ and ‘agree’) and disagreeing (sum of ‘strongly disagree’ and ‘disagree’). In the Spearman’s rank correlation analyses on these opinions, a 5-point scale from ‘strongly agree’ to ‘strongly disagree’ was used after excluding the more infrequent (0.4–4.8% depending on the statement) answer options ‘do not know’ or ‘prefer not to say’. Furthermore, multinomial logistic regression was used to analyze the influence of various factors on the BAC level category. As a reference category the ‘moderate’ BAC level (0.001–0.009%), containing the majority of the people, was used to compare factors influencing abstinence (BAC = 0), as well as being highly intoxicated (BAC ≥ 0.10%) compared to moderate drinking. Factors included in this analysis were either continuous (age) or transformed into dichotomous variables. Factors not included in the analysis were *occupation,* since it could not be transformed into a dichotomous variable, and *day* and *entrance* since the median BAC levels were not significantly different between the categories of these factors. Gender differences in alcohol use and drinking habits were analyzed using Pearson’s chi-squared test. The significance level was *p* ≤ 0.05.

## Results

### Sample characteristics

Of a total of 1633 festivalgoers who were invited to the study, 1410 agreed to participate, and 223 did not (dropout rate: 13.7%). The demographic characteristics of the participants show (Table 1) that the majority were male, ≤ 25 years old and working full-time. More specifically, the median age was 23 years (interquartile range: 20–28 years). There was a similar distribution of gender and estimated age among the people refusing participation (65.5% male, 34.5% female; median estimated age 25 years). Furthermore, most participants had a 4-day festival ticket including camping and were about to enter the festival area.

**Table 1:** Demographic characteristics

	% (n)
Gender	
Male	63.7 (898)
Female	34.9 (492)
Other	0.1 (2)
Age (years)	
16–17	4.9 (69)
18–20	23.1 (326)
21–25	37.5 (529)
26–30	15.2 (214)
≥ 31	19.0 (268)
Occupation	
No occupation	2.1 (29)
Working full-time	64.3 (906)
Working part-time	7.9 (111)
University	10.9 (154)
High school	7.0 (98)
Working & studying	7.4 (104)
Ticket type	
1-day	10.1 (142)
4-day	14.9 (210)
4-day with camping	74.5 (1050)
Entering	66.5 (937)
Exiting	32.4 (457)
Thursday	50.7 (715)
Friday	49.3 (695)
Main entrance	47.8 (674)
Camping entrance	52.2 (736)

Demographic characteristics of the 1410 participants are presented as percentages of all participants and in total numbers (n). Data is missing for the following variables and number of participants: gender (*n* = 18), age (*n* = 4), occupation (*n* = 8), ticket type (*n* = 8) and entering/exiting (*n* = 16)

### BAC levels among festival visitors

The proportion of drinkers, i.e., people with BAC levels > 0%, was 81.4% (*n* = 1148). Among the drinkers, the median BAC level was 0.082% (Table 2).

**Table 2:** BAC levels among different groups and factors

	BAC (%) of all participants (*n* = 1408)			BAC (%), participants with BAC > 0% (*n* = 1148)		
	median	(IQ range)	*p*-value	median	(IQ range)	*p*-value
Total	0.068	(0.019–0.110)		0.082	(0.046–0.120)	
Gender						
Male	0.076	(0.035–0.117)	< 0.001	0.085	(0.051–0.120)	0.002
Female	0.048	(0.002–0.094)		0.071	(0.033–0.110)	
Age (years)						
< 18	0.000	(0.000–0.058)	< 0.001	0.064	(0.039–0.089)	0.001
18–20	0.060	(0.018–0.102)		0.076	(0.042–0.111)	
21–25	0.071	(0.029–0.113)		0.081	(0.047–0.116)	
26–30	0.079	(0.034–0.124)		0.093	(0.055–0.131)	
≥ 31	0.068	(0.018–0.118)		0.086	(0.046–0.127)	
Occupation						
No occupation	0.068	(0.023–0.113)	< 0.001	0.082	(0.048–0.117)	< 0.001
Working full-time	0.074	(0.029–0.119)		0.087	(0.050–0.124)	
Working part-time	0.050	(0.000–0.100)		0.081	(0.041–0.122)	
University	0.066	(0.021–0.111)		0.084	(0.051–0.117)	
High school	0.030	(0.000–0.072)		0.064	(0.040–0.088)	
Working & studying	0.043	(0.006–0.080)		0.063	(0.027–0.100)	
Ticket type						
1-day	0.048	(0.002–0.093)	< 0.001	0.076	(0.045–0.107)	0.300
4-day	0.055	(0.002–0.109)		0.083	(0.046–0.121)	
4-day with camping	0.070	(0.026–0.114)		0.082	(0.045–0.120)	
Entering	0.070	(0.025–0.115)	< 0.001	0.084	(0.047–0.122)	0.007
Exiting	0.055	(0.010–0.100)		0.077	(0.039–0.115)	
Thursday	0.065	(0.016–0.115)	0.346	0.084	(0.046–0.123)	0.127
Friday	0.069	(0.028–0.111)		0.080	(0.048–0.123)	
Main entrance	0.068	(0.021–0.116)	0.453	0.085	(0.048–0.122)	0.087
Camping entrance	0.068	(0.024–0.112)		0.078	(0.042–0.115)	

Median and interquartile (IQ) range of blood alcohol concentration (BAC) levels are presented. Differences in BAC levels across the categories of gender, age, occupational activity, ticket type, status of entering/exiting, day of measurement and entrance were measured using the Mann-Whitney U-test or the Kruskal-Wallis test. Data is missing for the following variables and number of participants: BAC levels (*n* = 2), gender (*n* = 18), age (*n* = 4), occupation (*n* = 8), ticket type (*n* = 8) and entering/exiting (*n* = 16)

When the BAC levels of all participants (including BAC = 0) were analyzed, they differed significantly between groups in terms of gender, age, status of entering or exiting, and ticket type but not between day, hour, or entrance (Table 2, Mann-Whitney U-test or Kruskal-Wallis test). In brief, BAC levels were higher in males than in females, in those aged 26–30 years compared with other age categories, in participants working full-time compared with other occupation categories, in people entering versus exiting the festival, and in those holding a camping ticket versus other ticket types (Table 2). Among the drinkers (BAC levels > 0%), BAC levels also differed significantly across the same factors, except for ticket type. Similarly, BAC levels were highest among males, those aged 26–30 years, those working full-time, and those entering the festival.

**Fig. 1:**
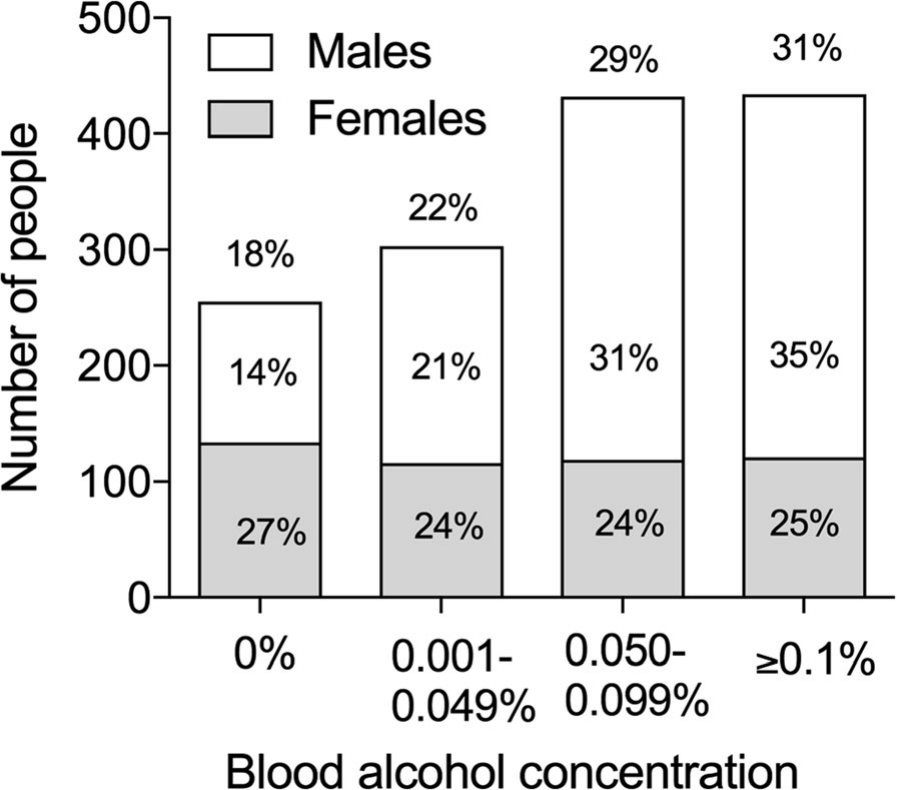
BAC level distribution across gender. Blood alcohol concentration (BAC) levels were measured using breath analyzers. BAC data were divided into the following categories: no alcohol (0) or low alcohol (0.001–0.049%) consumption, intoxication (0.050–0.099%) and high intoxication (≥0.10%). The data represent the distribution across these categories and are presented as numbers of people (bar) and percentages (above bars). This distribution across categories is also presented for each gender separately as numbers of people (bar) and percentages (within bars). Chi-squared tests showed a significant effect of the distribution of gender across BAC category (Χ^2^(4) = 49.64, *p* < 0.001). Z-tests revealed significant gender differences in all categories except the category 0.001–0.049%

The distribution across different BAC level categories is displayed in Fig. 1. For example, 31% of the respondents had BAC levels ≥0.10%, indicating high alcohol intoxication. There was a significant effect of gender on the distribution across BAC level categories (Fig. 1, Person’s chi-squared, Χ^2^(4) = 49.64, *p* < 0.001) and a significant difference between genders within each category (z-test), except the BAC level category 0.001–0.049%. For example, a higher number of females had BAC levels of 0 compared with males, and a higher number of males had BAC levels of ≥0.10% compared with females. Among visitors holding a 1-day or 4-day ticket without camping, 21.8 and 27.6%, respectively, had BAC levels above 0.10% compared with 33% of visitors holding a camping ticket (Person’s chi-squared, Χ^2^(6) = 39.92, *p* < 0.001).

Of the underage participants (16–17 years old, *n* = 69), 38 (55%) had no detectable BAC levels, 28 (41%) had BAC levels below 0.10%, and 3 (4%) had BAC levels above 0.10%. The median BAC level among the drinkers in this age category was 0.064%.

### Visitors report risky drinking habits

Participants self-reported drinking habits by answering the AUDIT-C questionnaire. The frequencies of alcohol use and binge drinking, the amounts of standard alcoholic drinks during a typical day, and the calculated frequencies of risky alcohol consumption are displayed in Table 3. Alcohol was consumed by 98% of the participants. The majority of the participants reported consumption of alcohol two to four times a month and stated that they drank five or more units per occasion. In addition, more than half of the participants reported binge drinking (≥ 6 units per occasion) at least once a month, while only 6% of participants reported to never binge drink. Risky alcohol use, determined by the calculated AUDIT-C score, was very frequent, independent of the cut-off used. Lastly, for each question or measurement, the distribution differed significantly across gender (Table 3, Pearson Chi-square test). Compared with females, males reported drinking alcohol more frequently and in larger amounts and reported binge drinking more often.

**Table 3:** Self-reported drinking habits across gender using AUDIT-C and calculated frequencies of risky alcohol consumption using 3 different cut-off levels

AUDIT-C	Male % (n)	Female % (n)	Total % (n)	Gender Χ^2^ (df), *p*-value
Question 1: Frequency of alcohol use				
Never	0.9 (8)	4.1 (20)^*^	2.0 (28)	49.99 (4), *p* < 0.001
Monthly	16.7 (150)	25.7 (126)^*^	19.9 (276)	
2–4 times a month	58.0 (520)	57.0 (280)	57.6 (800)	
2–3 times a week	21.2 (190)	12.4 (61)^*^	18.1 (252)	
≥4 times a week	3.2 (29)	0.8 (4)^*^	2.4 (34)	
Question 2: Reported alcohol use on a typical day				
≤4 units	23.5 (210)	47.4 (225)^*^	31.8 (435)	81.10 (1), *p* < 0.001
≥5 units	76.5 (682)	52.6 (250)^*^	68.2 (934)	
Question 3: Frequency of binge drinking (≥6 units)				
Never	2.7 (24)	12.6 (61)^*^	6.1 (85)	114.69 (4), *p* < 0.001
<Once a month	26.0 (233)	42.2 (205)^*^	31.7 (438)	
Monthly	47.2 (422)	33.3 (162)^*^	42.2 (584)	
Weekly	23.5 (210)	11.5 (56)^*^	19.4 (268)	
Daily or almost daily	0.7 (6)	0.2 (1)	0.5 (7)	
AUDIT-C score: Risky alcohol use				
> 4 for men, 3 for women	81.0 (726)	73.5 (361)^*^	78.4 (1087)	10.54 (1), *p* = 0.001
> 5 for men, 4 for women	68.5 (614)	53.8 (264)^*^	63.3 (878)	29.74 (1), *p* < 0.001
≥6 for men & women	68.5 (614)	37.1 (182)^*^	57.4 (796)	128.37 (1), *p* < 0.001

Frequencies in percent and number (n) of people across the categories of questions of the Alcohol Use Disorder Identification Test-Consumption (AUDIT-C) are shown. The frequencies of risky alcohol use according to different cut-offs used in research are presented. The effects of gender were analyzed using Pearson’s chi-squared test followed by the z-test

^*^Column proportions differ significantly (*p* < 0.05) between men and women. Data is missing for the following variables and number of participants: BAC (*n* = 2); AUDIT-C question 1 (*n* = 2), question 2 (*n* = 8) and question 3 (*n* = 11)

### Opinions on alcohol use and alcohol policies

Visitors at the festival were asked about their opinions on alcohol use and alcohol policies in relation to nightlife in general and in relation to the festival (statements S1–S6). The results (Table 4) show high support for alcohol policies regarding underage people and highly intoxicated people. For example, most participants agreed that drunk people should not be able to obtain more alcohol at the festival (S5) and that this measure would improve their nightlife experience (S6), including among those that were highly intoxicated (BAC ≥ 0.10). Furthermore, most people disagreed that highly intoxicated people should be able to enter the festival (S4) or that underage people should be able to obtain alcohol (S2). Less than one in four highly intoxicated people agreed that drunk people should be able to enter the festival area (S4).

**Table 4:** Opinions on alcohol use and their relation to BAC levels

	Overall sample		Within BAC category: 0% 0.001–0.09 > 0.10%			Within gender: Male Female		
	agree	disagree	agree	agree	agree	agree	agree	Χ^2^ (df = 1)
	% (n)	% (n)	% (n)	% (n)	% (n)	% (n)	% (n)	*p*-value
S1	29.3 (411)	48.3 (679)	13.5 (35)	29.7 (210)	37.8 (165)	31.6 (283)	24.7 (121)	7.35 *p* = 0.007
S2	15.2 (212)	73.2 (1031)	9.4 (24)	16.1 (113)	17.0 (74)	19.0 (168)	8.2 (40)	28.39 *p* < 0.001
S3	75.2 (1055)	9.3 (130)	82.2 (212)	75.9 (535)	70.3 (306)	70.8 (630)	82.9 (406)	24.60 *p* < 0.001
S4	19.8 (276)	63.7 (898)	15.2 (39)	19.8 (139)	22.6 (98)	24.2 (215)	11.9 (58)	29.74 *p* < 0.001
S5	89.0 (1250)	7.2 (102)	95.0 (245)	90.9 (643)	82.4 (360)	86.8 (777)	93.0 (455)	12.56 *p* < 0.001
S6	85.6 (1189)	7.0 (99)	87.6 (126)	86.3 (606)	83.2 (355)	83.5 (735)	89.6 (438)	9.37 *p* = 0.002

Opinions on alcohol use and alcohol policies were assessed through the following statements: S1) A good night out means getting drunk. S2) It is acceptable for people under 18 to buy or be bought alcohol. S3) Drunk people ruin a night out. S4) Drunk people should be able to enter the festival area. S5) People who are drunk should not be able to obtain more alcohol. S6) Not providing people who are already drunk with more alcohol would improve nights out. Frequencies of agreement are presented for the overall sample (left), within each blood alcohol concentration (BAC) level category (middle) and within each gender (right). Data represent the number of people (n) and percentage. The effects of gender were analyzed using Pearson’s chi-squared test

In contrast, opinions on alcohol use in relation to the nightlife experience varied among individuals. Although most people, including being highly intoxicated themselves, agreed that drunk people ruin a night out (S3), agreeing to the statement that a good night out means getting drunk (S1) varied considerably among individuals and BAC levels. In fact, there was a significant positive correlation between level of agreement (5-point scale) to S1 and BAC levels (Spearman r = 0.227, *p* < 0.001). Except for S6 the other statements were also significantly correlated with BAC levels but to a negligible degree (S2: Spearman r = 0.079, *p* < 0.01, S3: Spearman r = − 0.089, *p* < 0.01, S4: Spearman r = 0.070, *p* < 0.01, S5: Spearman r = − 0.093, *p* < 0.001).

Compared with women, men were significantly less supportive of restrictive alcohol policies and more liberal towards alcohol intoxication (Table 4), shown by more men than women agreeing with statements S1, S2, and S4, and fewer men than women agreeing with statements S3, S5, and S6.

### Prediction of BAC levels among visitors

To explore factors that could influence BAC levels, multinomial logistic regression was performed using BAC level category (0; 0.001–0.09; ≥ 0.10%) as the dependent variable and the following independent variables: age, gender, occupation, ticket type, reported risky alcohol use (AUDIT-C threshold of 4 for women and 5 for men), entering versus exiting, and agreeing with statements S1–S5. Statement 6 was not included in the analysis due to a high correlation with statement 5 (Spearman r = 0.68, *p* < 0.001).

The model was overall significant (Χ^2^(22) = 203, *p* < 0.001) and had a pseudo R-square of 0.16 (Nagelkerke). The following factors were overall significant: age (Χ^2^ = 21.16, *p* < 0.001), gender (Χ^2^ = 23.67, *p* < 0.001), ticket type (Χ^2^ = 29.41, *p* < 0.001), reported risky alcohol use (Χ^2^ = 37.29, *p* < 0.001), entering (Χ^2^ = 21.20, *p* < 0.001), S1 (Χ^2^ = 18.87, *p* < 0.001), and S5 (Χ^2^ = 13.12, *p* = 0.001).

The contributions of these factors to BAC level were analyzed by comparing BAC levels of 0% and ≥ 0.10% to the reference category 0.01–0.09% (Table 5). Being female was significantly associated with a higher chance of having a BAC level of 0. In contrast, having risky alcohol use habits, possessing a camping ticket, entering the festival or agreeing with S1 (‘A good night out means getting drunk’) were all associated with a lower chance of having a BAC level of 0. Having risky alcohol use habits increased the risk of having a BAC level above 0.10%, whereas agreeing with S5 (‘People who are drunk should not be able to obtain more alcohol’) decreased it.

**Table 5:** Factors influencing undetectable and high alcohol intoxication levels

D.V. BAC 0.001–0.09% vs:	0%				≥0.10%			
	χ^2^	O.R.	95% C.I.	*p-value*	χ^2^	O.R.	95% C.I.	*p-value*
Age	6.97	0.97	0.95–0.99	0.008	7.70	1.03	1.00–1.04	0.006
Gender (female vs. male)	16.38	1.93	1.41–2.67	< 0.001	2.16	0.81	0.61–1.07	0.142
Risky alcohol use	17.78	0.49	0.35–0.68	< 0.001	8.96	1.58	1.17–2.12	0.003
Camping ticket	16.19	0.37	0.23–0.60	< 0.001	1.74	1.38	0.86–2.23	0.187
Entering the festival	12.84	0.53	0.38–0.75	< 0.001	3.40	1.30	0.99–1.74	0.065
Agree with S1	10.15	0.49	0.32–0.76	0.001	3.57	1.31	0.99–1.74	0.059
Agree with S2	0.55	0.82	0.49–1.38	0.457	0.001	0.99	0.70–1.41	0.973
Agree with S3	1.39	1.28	0.85–1.93	0.238	1.41	0.84	0.62–1.12	0.235
Agree with S4	0.15	1.09	0.70–1.71	0.695	0.93	0.85	0.61–1.19	0.335
Agree with S5	0.68	1.34	0.67–2.70	0.408	10.20	0.53	0.36–0.78	0.001

Using multinomial logistic regression analysis, the influence of the following factors on blood alcohol concentration (BAC) levels were investigated: age, gender, self-reported risky alcohol use according to AUDIT-C (cut-off 4 for women, 5 for men), ticket type (camping ticket vs. 4-day or 1-day ticket without camping), entering versus exiting the festival, and agreeing with statements S1 to S5 (*n* = 1318). BAC categories of 0% and ≥ 0.10% were compared with the reference category 0.001–0.09%. All shown independent variables are dichotomous except for age (continuous)

## Discussion

This study investigated the level of alcohol intoxication among visitors at one of the largest music festivals in Sweden. The study participants were asked about their alcohol drinking habits and opinions on alcohol policies in relation to the festival, and their BAC levels were measured through breath analysis. Eighty-two percent of participants had a positive BAC level, with a median of 0.082%. Thirty-one percent of participants had a BAC level ≥ 0.10%. The majority of people reported risky alcohol consumption habits. Compared with females, males had higher BAC levels and more frequently reported risky alcohol consumption. Furthermore, there were high levels of support for restrictive alcohol policies despite high alcohol consumption levels.

The majority (82%) of the people had detectable BAC levels and therefore, had been drinking, similar to most studies in festivals and clubs [33–36] but to a higher extent than at the EDM festival in Miami (US) where only 40% had BAC levels above 0 [32]. The average BAC level of 0.082% was somewhat lower than most studies conducted in festivals, large events and clubs, which reported an average of 0.10% [32–35]. Nevertheless, in the present study about half of the participants had BAC levels above the UK and US DUI limit of 0.08% and about one third had levels above 0.10%, the DUI level considered to be a gross crime in Sweden. These frequencies are higher compared with a study of visitors at clubs in San Francisco, US [36]. Together, the average BAC level was still rather high and a significant number of people were highly intoxicated. In addition, 31 underage people had an average BAC level of 0.064%. Therefore, these results call into question whether the Swedish law was observed, as the law does not permit obviously intoxicated people to be allowed entrance to a licensed premise or allowed to remain there, and it does not permit serving alcohol to underage people.

BAC levels were higher for people entering than for people exiting the premises, indicating a greater problem of preloading compared with drinking inside the festival area. Therefore, future interventions might need to focus on increasing the frequency of denying entry to highly intoxicated individuals.

We hypothesized that many people consume large amounts of alcohol in the camping area. The finding that BAC levels at both the main entrance and camping entrance were similar is probably due to the fact that many people who used the main entrance possessed a camping ticket (over 50%). However, the median BAC levels were significantly higher among people with a camping ticket but only if all participants were included in the analysis (not only those with detectable BAC levels). Furthermore, the multinomial analysis showed that a camping ticket decreased the likelihood of having a BAC level of 0% but did not influence the risk of having a BAC level of ≥0.10%. These results indicate that more people who did not have a camping ticket had a BAC level of 0% rather than that people with a camping ticket were drinking significantly more in general. One explanation may be that many people without a camping ticket commuted by car and therefore did not drink, as the legal limit for BAC level while driving is low in Sweden (BAC of 0.2‰ ≈ 0.02%). Nevertheless, approximately one in five and one in four of those holding a 1-day or 4-day ticket without camping, respectively, had BAC levels above 0.10%, and the police reported 10 cases of DUI.

In the present study, more than half of the participants reported binge drinking (6 or more units) at least monthly, and almost 20% reported binge drinking weekly or more often. In comparison, previous studies at Australian festivals reported that about 30 % of visitors binge drink weekly [29, 30]. Furthermore, the majority of participants in the present study reported risky alcohol consumption: 63 and 78% respectively using a cut-off of 4/5 or 3/4 for women/men. The frequency of risky alcohol consumption was even higher among visitors at a Swedish EDM event: 78% or 90% using the 4/5 or 3/4 cut-off, respectively [33]. Although slightly different measurements and cut-offs have been used, Swedish surveys report similarly high levels of binge drinking and risky alcohol consumption among young people [59, 60]. Moreover, in the present study, risky alcohol consumption was associated with BAC levels ≥0.10%, similar to previous studies on visitors at an EDM event [33]. In line with this, several previous studies have demonstrated that self-reported drinking habits in students and young people predict high BAC levels during an event [36, 60, 61].

Self-reported drinking habits differed significantly between genders. Men used alcohol and engaged in binge drinking more frequently than women. Furthermore, the frequency of risky alcohol consumption was higher in men than in women. A study among Belgian club- and festivalgoers also reported that men drank more frequently than women [28]. In addition, a study of young adults who attended New York night clubs showed that self-reported binge drinking was frequent and that more men than women reported binge drinking [11]. However, there were no significant differences between genders regarding risky alcohol consumption in the Swedish EDM event study [33].

In the present study, there was also a significant gender difference in measured BAC levels, indicated by differences in median BAC levels and frequencies across BAC categories. Significantly more women than men had BAC levels of 0. Multinomial logistic regression indicated that being female increased the odds of having a BAC level of 0 but did not change the odds of a high BAC level (≥ 0.10%) versus a low to moderate BAC level. Nevertheless, significantly more men than women had a BAC level ≥ 0.10%, similar to the previous study of visitors at a Swedish EDM event [33]. In contrast, neither the study in Norwegian clubs [34] nor the study in clubs in San Francisco found any effect of gender on BAC levels.

Pursuant to Swedish law, licensed premises must ensure that alcohol is not sold to underage people (i.e., < 18 years old) or to people who intend to give it to underage people. While it would have been easy for serving staff to comply with the former due to the color-coded bracelets, the latter would have been harder to avoid, as the large festival area was difficult to oversee. Of the 69 underaged people in our study, 45% had detectable BAC levels (median 0.064%), indicating that they could easily obtain alcohol inside or near the festival area. This result is cause for concern and calls into question the appropriateness of allowing underage people into the festival area.

There was generally high support for restrictive alcohol policies among the participants, similar to previous Swedish studies regarding alcohol intoxication in clubs [62] or soccer arenas in Sweden [63]. Strong public support for restrictive alcohol policies has also been demonstrated in the US and Canada [64, 65]. In the present study, restrictive alcohol policies regarding highly intoxicated people were mostly supported even by those with high BAC levels of ≥0.10%, which raises the question if they considered themselves to be highly intoxicated. Nevertheless, there was a small effect of decreasing support for restrictive alcohol policies with increasing BAC levels.

Regarding alcohol use affecting the nightlife experience, the majority of people considered that highly intoxicated people disturb their nightlife experience, even among those with BAC ≥ 0.10, which further supports the doubt that these visitors consider themselves to be highly intoxicated. Furthermore, BAC levels correlated positively with agreeing to that getting intoxicated is important for a good nightlife experience.

Regarding both opinions on alcohol use and alcohol policies, men were more liberal towards alcohol use and less supportive of restrictive alcohol policies compared to women. In a study investigating opinions on alcohol drinking in soccer arenas, men had a more liberal attitude than women about alcohol use in the arenas, although there was no significant effect of gender regarding opinions on policies towards highly intoxicated individuals [63]. In contrast, similar to the present study, a previous study on alcohol drinking in clubs in Sweden demonstrated that men were less supportive of restrictive alcohol policies than women [62]. Overall, since the level of alcohol intoxication was higher among men, risky alcohol consumption habits were more frequent among men and men were less supportive of restrictive alcohol policies, interventions could be developed further by considering these gender effects.

### Strengths and limitations

One strength of the present study is the use of biological sampling for measuring alcohol intoxication as opposed to self-reports. This approach improves the validity of our results, as the data are not biased by under- or overreporting. Other strengths of the study are the large sample size (1410 participants), the high response rate (83%), the collection of data from both entrances to/exits from the festival area, and the randomized recruitment of participants. Thus, we believe that our data are representative of all visitors attending the festival.

One limitation of the present study is that it was conducted at just one festival. Alcohol intoxication levels at several music festivals should be investigated in order to increase generalizability. Moreover, the results presented herein might not be generalizable to music festivals in other countries. Another limitation is that approximately half of the participants had BAC levels above 0.08%, which may have affected their cognitive functioning, including memory, and, therefore, influenced their answers to other questions. Furthermore, the data on alcohol drinking habits relied on self-reporting, which can be subject to underreporting and underestimation. We did not record if the individuals participate in the study belonged to a group or not and it is, therefore, unknown if group size introduced a selection bias.

## Conclusions

High levels of alcohol intoxication among visitors at music festivals are problematic and are associated with violence, injuries, drunk driving, sexual risk-taking and sexual assaults. The present study demonstrates high levels of alcohol intoxication together with high support for restrictive alcohol policies among visitors at one of Sweden’s largest music festivals. Males had higher levels of alcohol intoxication and were less supportive of restrictive alcohol policies compared to females. Risky alcohol consumption habits predicted heavy alcohol intoxication at the festival. These findings indicate both a need and support for alcohol preventive strategies being implemented at festivals to reduce the frequency of highly intoxicated individuals. Tailoring our previously developed model, which includes community mobilization, training, and improved enforcement, to music festivals is a promising approach.

## Abbreviations

AUDIT-C: Alcohol Use Disorder Identification Test- Consumption
BAC: Blood alcohol concentration
STAD: Stockholm prevents alcohol and drug problems
